# Yttrium-90 radioembolization for treatment of anaplastic meningioma liver metastases

**DOI:** 10.1016/j.radcr.2025.09.042

**Published:** 2025-11-11

**Authors:** Mohammad-Kasim Fassia, Arsalan Haghdel, Fred Pelzman, Rajiv Magge, Susan Pannullo, Benjamin Liechty, Theodore H. Schwartz, Jonathan P.S. Knisely, Jana Ivanidze, Brian Currie

**Affiliations:** a Department of Radiology, Weill Cornell Medicine, New York, NY, USA; b Department of Medicine, Weill Cornell Medicine, New York, NY, USA; c Department of Radiation Oncology, Weill Cornell Medicine, New York, NY, USA; d Department of Neurological Surgery, Icahn School of Medicine, Mt. Sinai Hospital, New York, NY USA; e Department of Biomedical Engineering, Cornell University, Ithaca, NY; f Department of Diagnostic Radiology, Geisinger Health, Wilkes-Barre, PA, USA

**Keywords:** Transarterial radioembolization, ablation, DOTATATE PET-CT, Meningioma, WHO III meningioma

## Abstract

Meningiomas are the most common primary intracranial neoplasm. The WHO classification, focusing on histomorphologic and molecular features, distinguishes WHO grade -1, -2, and -3 tumors. WHO-3 meningiomas are exceedingly rare, comprising 1%-3% of cases, and have the propensity to metastasize to the lungs, followed by bone and liver. Due to their rare occurence, there are no consensus guidelines on metastatic meningioma management. External beam radiation therapy is often the treatment of choice, therefore transarterial radioembolization is a reasonable option for meningioma metastases to the liver. In this case report, we describe the clinical course and outcomes of a patient with WHO-3 meningioma and extensive hepatic metastases which were initially detected during workup for possible renal transplant, confirmed with DOTATATE PET/CT, and successfully treated with Yttrium-90 radioembolization and ablation.

## Case presentation

Meningiomas are the most common primary intracranial neoplasm, classified based on histomorphologic and molecular features into WHO grades 1-3 (WHO 1-3). While WHO-3 meningiomas are rare, comprising only 1%-3% of cases, they are clinically significant given their higher likelihood of recurrence even after surgical resection and radiation therapy (RT), and their propensity to metastasize [[1], [2], [3]]. Patterns of metastasis in WHO-3 meningiomas show the lungs are the most common location, followed by bone and liver. As meningiomas across all WHO grades express high levels of somatostatin receptor 2 (SSTR2), DOTATATE PET/CT and PET/MRI have emerged as a valuable adjunct modality in meningioma staging and management [[4], [5], [6], [7], [8]]. Given its rare occurence, treatment guidelines for metastatic meningiomas are not well defined. Previous case reports have investigated chemotherapy, resection, and/or radiation therapy as possible treatment options [9]. Meningiomas are highly vascular and radiosensitive tumors therefore, interventional radiology (IR) embolization represents a possible therapeutic approach in meningiomas metastatic to the liver. In addition, radioembolization selectively targets a lesion allowing for higher radiation dose and fewer systemic side effects when compared to radiation therapy or chemotherapy [10,11]. Despite the numerous benefits, there are a few drawbacks to radioembolization such as liver failure, especially in the setting of poor baseline liver function or large liver tumor burden and renal failure in the setting of end-stage renal disease considering Yttrium-90 microspheres are renally excreted. At baseline, the patients liver function was within normal limits and he was a candidate for renal transplant, therefore his health care management team felt that the benefits from transarterial radioembolization outweighed the risks from the procedure. In this case report, we describe the clinical course and outcomes in a patient with WHO-3 meningioma and extensive hepatic metastases which were diagnosed using DOTATATE PET/CT and successfully treated with Yttrium-90 radioembolization and ablation.

**Fig. 1:**
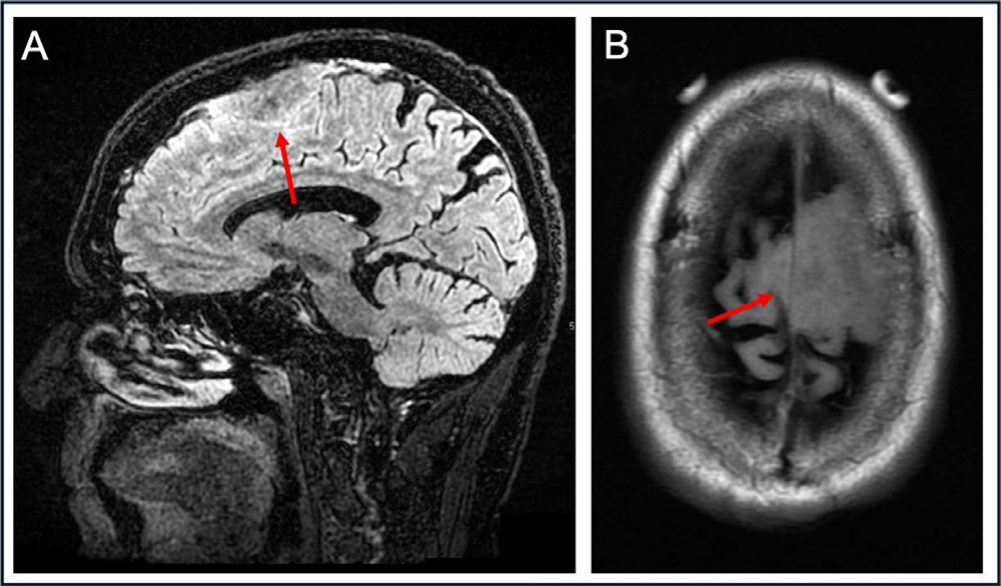
Preoperative sagittal T2 FLAIR (A) and axial (B) 3D T1 MRI of the brain. Overlying the left frontal convexity, there is an extra-axial mass which is mildly T2 hyperintense, crosses midline to the right hemispheric convexity, and invades the adjacent superior sagittal sinus (red arrows). The lesion measured approximately 5.4 × 4.2 × 2.0 cm (transverse, anterior-posterior, and cranial-caudal dimensions). The patient was planned for bifrontal craniotomy for removal of the dural-based lesion 2 weeks after identification on MRI. Pathology revealed a WHO grade 1 meningioma with invasion of the superior sagittal sinus. The patient did not receive intravenous contrast preoperatively due to concerns of impaired renal function.

A 75 year old man with a history of hypertension, coronary artery disease, and chronic kidney disease presented to our Emergency Department for evaluation of new onset aphasia and gait abnormality. A noncontrast head CT revealed no acute intracranial abnormalities, however a dural based lesion overlying the left frontal convexity was identified. A subsequent noncontrast brain MRI confirmed the extra-axial, dural based mass measuring up to 5 cm with invasion of the superior sagittal sinus (Fig. 1).

Two weeks after presenting to the ED, the patient underwent a bifrontal craniotomy for subtotal resection/debulking of the dural-based neoplasm (with residual tumor in the superior sagittal sinus, which was not amenable to resection). Pathology demonstrated WHO-1 meningioma, therefore following resection, monitoring with serial imaging was favored over adjuvant radiation therapy. Two years after resection, the patient presented with worsening headaches, shaking spells and dysfluency. A follow-up MRI demonstrated meningioma recurrence in the prior resection cavity.

**Fig. 2:**
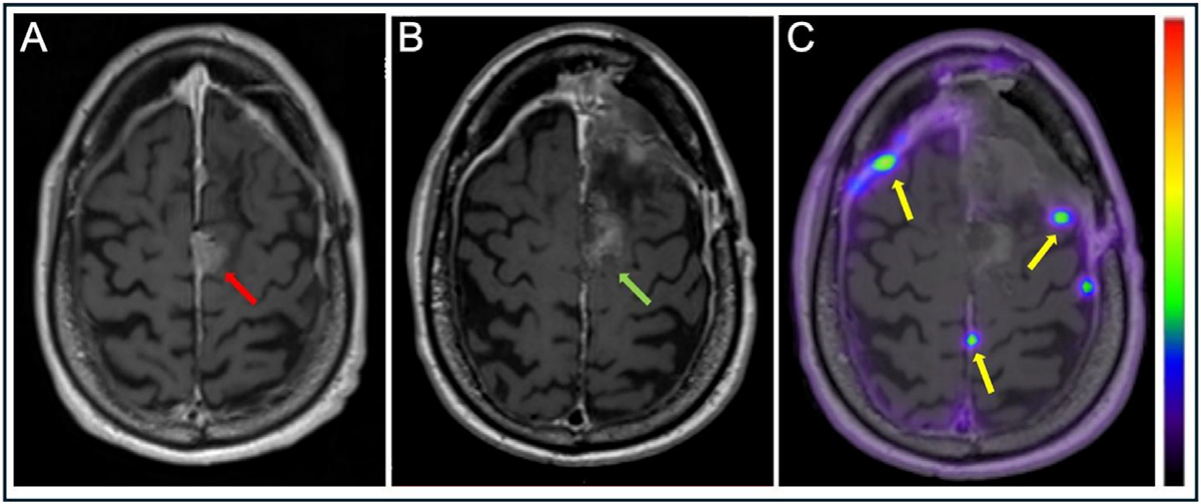
Axial T1 with contrast prior to second craniotomy (A). Overlying the left and right frontal convexity, there is an extra-axial, avidly enhancing, dural based lesion in the prior resection cavity (red arrow). The dural-based lesion caused mass effect on the subjacent brain parenchyma. Following MRI, the patient underwent resection of the recurrent dural-based mass as seen on postoperative axial T1 with contrast image (B). A focal area of enhancement in the resection cavity was suspicious for residual tumor or postoperative changes (green arrow). Pathology revelead a WHO grade III meningioma, suggesting progression of the meningioma. Postoperative axial T1 weighted MRI with contrast fused with DOTATATE PET (C, windowed SUV 0-15) demonstrates multiple residual, lesions along the dura (yellow arrows) that were not previously visualized on prior MRI with contrast.

**Fig. 3:**
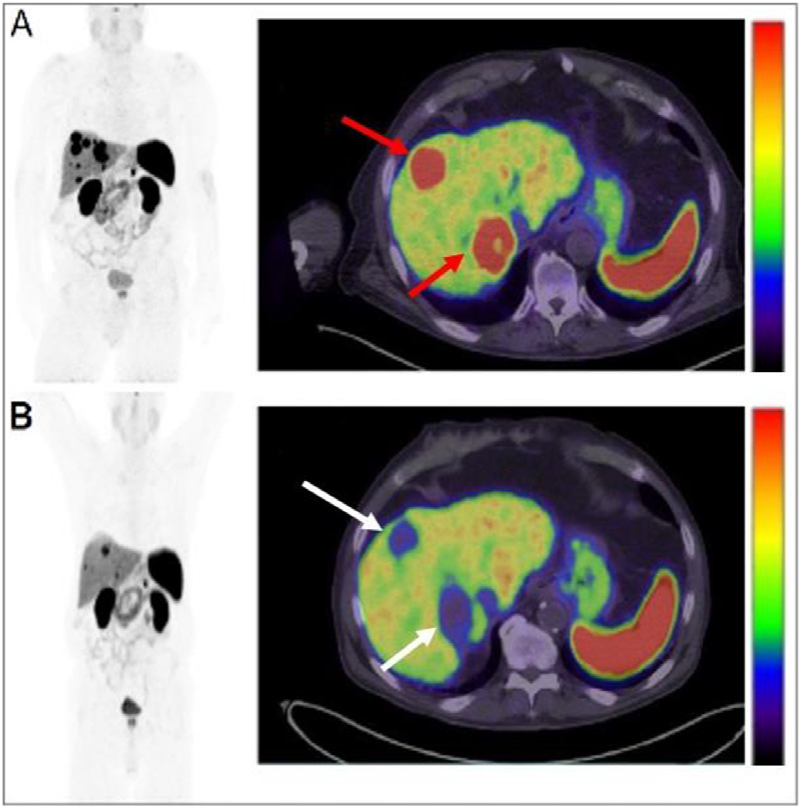
Whole-body Maximum intensity projection (MIP) image and axial fused DOTATATE PET/CT image at the level of the liver and spleen, windowed 0-15 SUV prior to radioembolization (A) demonstrate multiple DOTATATE avid hepatic lesions (red arrows on axial fused image; also evident on MIP image). The segment 4 and 7 lesions were mapped, selectively catheterized, and treated with Y90 TheraSpheres at a dose of 300 Gy. Following radioembolization, MIP and axial DOTATATE PET/CT (B) show complete resolution of DOTATATE avidity in the previously identified hepatic metastases, consistent with complete response to radioembolization therapy (white arrows). Few smaller persistent hepatic metastases, as evident on the MIP image, were not targeted by Y90 therapy.

A second bifrontal craniotomy was performed to resect the recurrent meningioma and pathology revealed a WHO-3 meningioma based on mitotic activity and homozygous deletion of CDKN2A detected by Oncomine sequencing, which also revealed a pathogenic NF2 c.557+2T>G splice mutation (thus indicating secondary progression from a WHO-1 tumor). The immediate postoperative MRI scan documented a possible small-volume of residual meningioma along the right parasagittal falx that was felt to possibly represent postsurgical change or minimal residiual tumor. Despite resection of the recurrent meningioma, the patient still experienced episodes of aphasia, leg shaking, and confusion following discharge. A referral for a radiation oncology consultation was placed, and a DOTATATE PET/MRI was ordered which revealed multiple dural-based, enhancing, DOTATATE avid lesions which were previously not identified on contrast enhanced MRI alone (Fig. 2). A total of 9 new lesions were identified, and confirmed the presence of a substanial postoperative residual tumor that was only suspected on the postoperative MRI. To treat these new lesions not previously identified on MRI, the patient received radiosurgery to the DOTATATE-PET identified meningiomas for a total of 3,500 cGy in 5 fractions. The patient did not suffer any complications following radiosurgery, however the patient did report worsening bilateral leg weakness. A follow-up MR following the second craniotomy and radiosurgery demonstrated stable intracranial meningioma without evidence of progression. Given the patient’s end-stage renal disease, he was concurrently evaluated for possible renal transplant, which included a preoperative renal CT abdomen and pelvis demonstrating multiple hepatic lesions without a known primary malignancy. Given the propensity of WHO-3 meningiomas to metastasize, a follow up skull base to thigh DOTATATE PET CT was performed which revealed multiple intensely DOTATATE avid lesions in the liver consistent with metastatic meningioma (Fig. 3). An ultrasound-guided biopsy of one of the DOTATATE avid hepatic lesions confirmed metastatic meningioma. No other extracranial disease sites were identified on DOTATATE PET/CT. A confirmatory DOTATATE PET/MRI of the brain showed response in the meningiomas previously treated with radiosurgery (decreased size and decreased DOTATATE avidity), and few new small foci of DOTATATE avidity were identified representing new meningiomas. These were felt amenable to further radiosurgical treatment. Given the number and distribution of hepatic lesions, intravascular radiotherapy with yttrium-90 (Y-90) glass beads were selected for hepatic metastases treatment.

## Methods

Hepatic metastases arterial mapping was performed with Technicium-99m Macro-aggregated albumin (Tc99m-MAA). An accessory left hepatic artery extending from the proximal gastroduodenal artery was noted on arterial mapping. The proper hepatic artery perfused metastasic lesions in segment 7 and segment 8, while the accessory left hepatic artery perfused the segment 4 and segment 8 lesions. Subsequent scintigraphy and SPECT/CT demonstrated 11.8% shunting from the hepatic artery to the lungs without evidence of significant extrahepatic activity. The single compartment model was selected over a multicompartment model because of the relatively small tumor volumes and desire for homogenous ablative dose given the hetereougenous distribution of liver lesions.

**Fig. 4:**
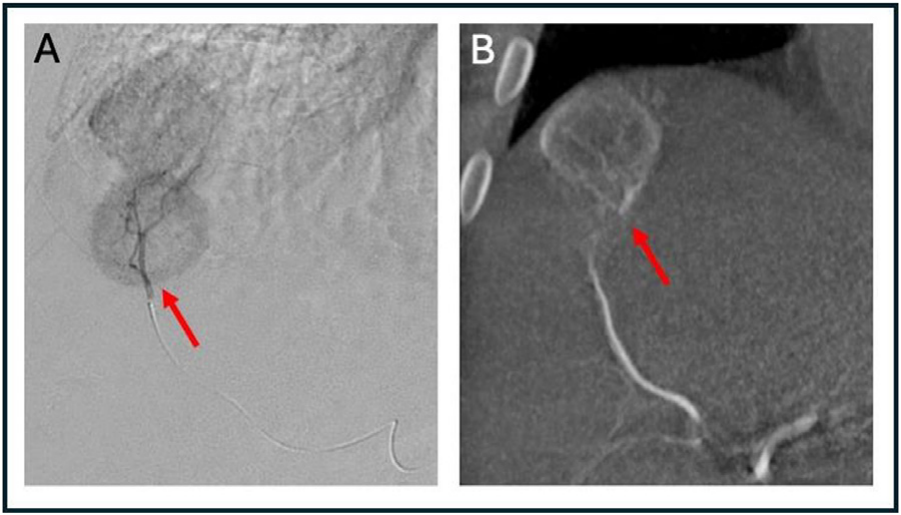
A selective hepatic arteriogram (A) demonstrates arterial supply to the segment 7 metastatic lesion subjacent to the hepatic capsule (red arrow). A Cone beam CT (B) confirms selective perfusion of the segment 7 lesion (red arrow). Minimal arterial enhancement is identified within the background parenchyma suggesting no variant anatomy or anomalous arterial supply. Following arterial mapping, this lesion was treated with Y90 TheraSpheres.

For radioembolization delivery, the segment 7 artery was catheterized via the proper hepatic artery and a radio-ablative dose of Y-90 TheraSpheres was administered with single-compartment dosimetry and target dose of 300 Gy (Fig. 4). Next, the segment 8 hepatic artery was catheterized via the proper hepatic artery and a second radioablative dose was administered, again with a target dose of 300 Gy (single-compartment). Initially, the patient reported no symptoms or complications after radioembolization. Four days following the procedure, however, the patient did reported low-grade fever, chills, and emesis which was treated with tylenol. His symptoms resolved several days following his procedure consistent with postembolization syndrome.

Following recovery from initial treatment, the patient returned for staged treatment of the Segment 4/8 lesions. The accessory left hepatic artery arising from the gastroduodenal artery was accessed, which was shown to feed the segment 4 and segment 6 metastases. Two separate aliquots of Tc-99m MAA (2.5 mCi each) were injected from the segment 4A and 4B arteries. Subsequent scintigraphy and SPECT demonstrated 16.3% lung shunting. Ablative doses were given to subsegmental volumes in Segments 4A and 4B based on the selective cone beams; these were dosed to 400 Gy (single compartment model). The patient tolerated the procedure without complication.

**Fig. 5:**
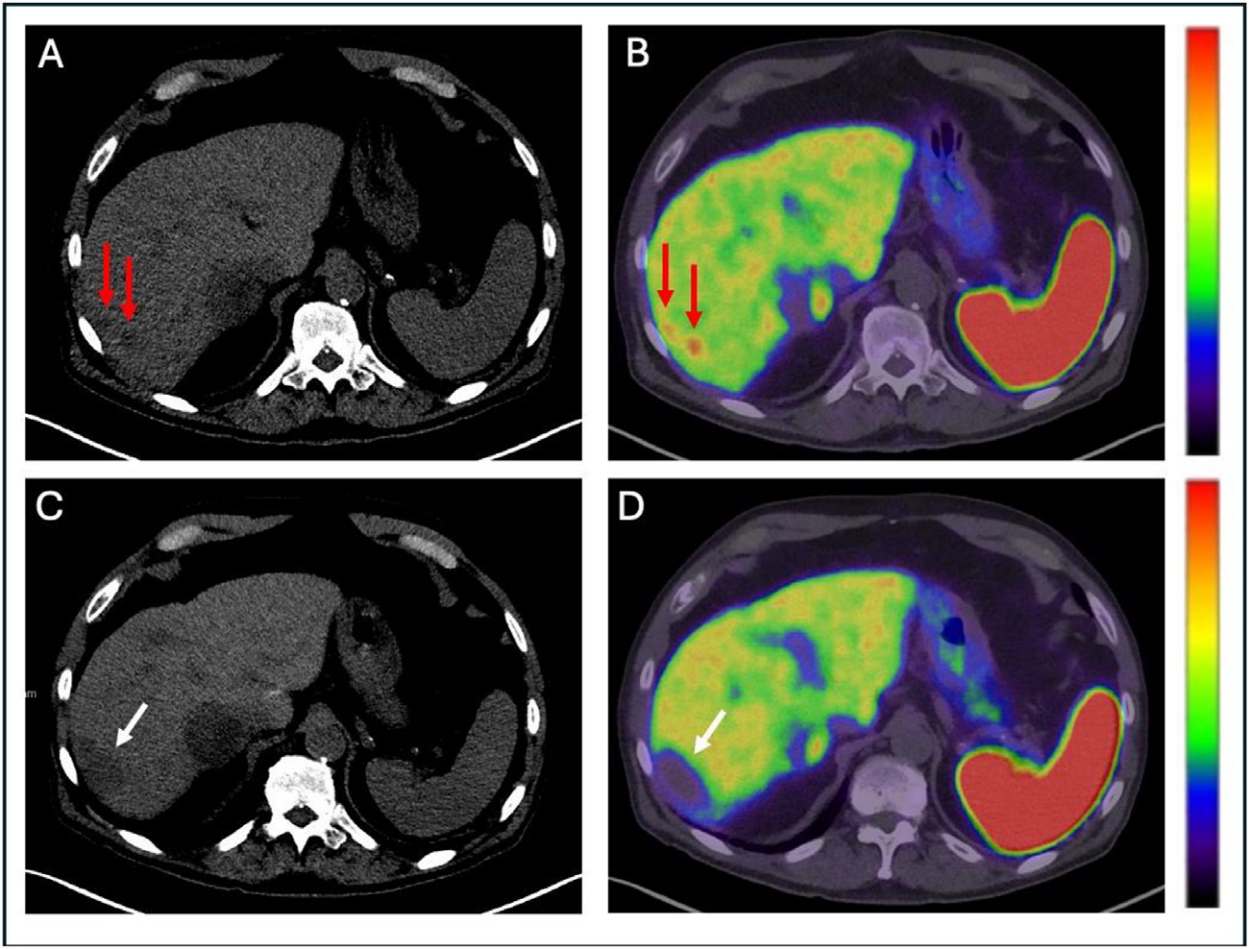
Axial abdominal CT (A) and corresponding fused DOTATATE PET-CT (windowed 0-15 SUV) (B) demonstrate 2 DOTATATE avid segment 6 lesions (red arrows) suspicious for recurrent metastatic meningioma. Two separate Neuwave PR15 XT probes were used to treat the Segment 6 lesions. Hydrodissection with saline was performed to insulate the abdominal wall. The settings for each ablation were 65 watts, for a total duration of 5 minutes. Axial abdominal CT (C) and fused DOTATATE PET-CT (D, windowed to 0-15 SUV) after thermal ablation demonstrate no DOTATATE avidity (white arrow), consistent with complete response. The combined ablation cavity was approximately 4 cm in diameter.

Additional ablative Y90 treatments were performed on the Segment 2 and 8 lesions 2 months after the second Y90 treatment (Fig. 5). Both metastatic lesions were dosed to 400 Gy using the single-compartment model given their small size (approximately 30 mL). A single treatment with Y90 was considered for this patient, however it may have exposed a significant portion of normal liver parenchyma to radiation given the patients variant arterial anatomy, scatter distribution of lesions, and low tumor-to-normal tissue (T/N) ratio. The patient also confirmed a strong interest in maximal therapy, therefore he was willing to undergo multiple treatments.

After two Y90 treatments, the previously identified Segment 6 metastases on the original DOTATATE scan had increased in size. Given the patient’s multiple Y90 treatments, the remaining Segment 6 metastases were treated with ablation. Two separate Neuwave PR15 XT probes were used to treat 2 lesions in Segment 6. Given the peripheral location of these lesions (subcapsular), hydrodissection with saline was performed to insulate the abdominal wall. The settings for each ablation were as follows: 65 watts, for a total duration of 5 minutes. The combined ablation cavity was approximately 4 cm in diameter. The patient was noted to have urine retention following the procedure, however it resolved shortly thereafter following straight catheterization.

### Outcome

Initial DOTATATE PET/CT demonstrated a total of 7, intensely enhancing hepatic lesions of varying size and avidity, with most lesions located in the right hepatic lobe (Table 1). Five lesions were identified in segment 7/8 and 2 lesions in segment 4A/4B. DOTATATE PET-CT after the initial radioembolization demonstrated markedly decreased SUV in 86% (6/7) of the hepatic lesions (summary of SUV changes presented in Table 1, representative images in Fig. 3).

**Table 1:** Table of each metastatic lesion within the liver.

Lesion location	Initial PET-CT	First PET-CT Post-Y90 treatment	Second PET-CT Post Y90 treatment
Segment 7 (anterolateral)	3.2 × 2.3 cm SUV 32.6	3.4 × 2.1 cm SUV 6.9	3.4 × 2.1 cm SUV 3.6
Segment 4, (anteromedial)	2.3 × 2.1 cm SUV 29.4	2.6 × 1.8 cm SUV 21.3	2.8 × 1.4 cm SUV 10.5
Segment 7 (anterior)	3.1 × 3.0 cm SUV 32.7	3.4 × 3.0 cm SUV 9.0	3.4 × 3.0 cm SUV 6.3
Segment 7 (posteromedial)	5.4 × 4.1 cm SUV 31.5	5.2 × 2.5 cm SUV 6.8	5.2 × 2.5 cm SUV 5.6
Segment 7 (posterolateral)	2.1 × 0.7 cm SUV 16.6	1.7 × 1.4 cm SUV 17.4	2.0 × 0.8 cm SUV 9.3
Segment 7 (posterolateral)	2.4 × 1.2 cm SUV 30.8	1.9 × 0.8 cm SUV 12.9	1.6 × 1.0 cm SUV 10.0
Segment 4 (anterior)	1.9 × 1.2 cm SUV 35	2.5 × 1.0 cm SUV 12.5	2.5 × 1.0 cm SUV 11.2
Segment 2 (posteromedial)			3.3 × 1.3 cm SUV 17.3

Each row contains a different metastatic lesion within the liver and each column represents the PET-CT from where the measurements were obtained. Each cell contains the size and avidity of the lesion at the time of the PET-CT.

Following treatment with Y90, 2 new focal areas of DOTATATE avidity within segments 1 and 2 were identified, and persistent activity in the segment 7 lesion, were suspicious for new and persistent hepatic metastases, respectively.

On the 1-year follow-up DOTATATE scan, there were some hepatic metastases with increased DOTATATE avidity and others that were unchanged from prior imaging (Table 1). For example, the previously treated segment 2 lesion showed increased size and peripheral avidity. In addition, the previously treated segment 7 lesion showed increased DOTATATE avidity. These findings were consistent with recurrent hepatic metastases. Clinically, the patient’s liver function, as measured by bilirubin, ALT, and AST, was unchanged following his procedures. The patient’s hepatic metastases were incidentally identifed during workup for renal transplant, and subsequently the patient’s renal function as measured via creatinine was also unchanged. Lastly, the patient’s lower extremity weakness persisted despite treatment most likely due to his unchanged intracranial disease following craniotomy and radiosurgery. Given his recurrent metastases, the patient is no longer a candidate for renal transplant and has elected for palliative therapy.

## Discussion

Metastatic meningioma is a rare condition with limited management guidelines in the literature, particularly as it relates to hepatic locoregional therapy. Metastatitic meningiomas can be treated with surgery, radiation therapy, or systemic chemotherapy. Each choice has its favorable characteristics and limitations. For example, surgey can adequately remove a lesion, however if a patient cannot tolerate anesthesia or if the lesion is in an unfavorable location (e.g. portal vein involvement) surgery may not be an option. Radiation therapy can be precise in treatment of metastatic meningioma but unfortunately there is increased risk for radiation toxicity as the size and number of metastatic lesions increases. Lastly, chemotherapy is another option, however prior studies have shown limited effectiveness [12]. An emerging treatment option with includes immunotherapy such as pembrolizumab [13] which has shown promising results for short-term periods. However, in this patient’s particular case, given the anatomic distribution of the hepatic lesions and their inherent hypervascularity, intra-arterial therapy was the most reasonable option. Transarterial radioembolization poses a risk for hepatotoxicity if excessive radiation is delivered to normal liver parenchyema, however the patient had preserved liver function, no predisposing conditions (HBV, HCV, etc), and no prior biliary interventions, therefore making him an excellent candidate for transarterial radiation embolization. For these reasons, we elected to pursue Y90 therapy for treatment of metastatic meningioma liver lesions.

Theraspheres were chosen as the Y90 vehicle due to operator preference and desire for less embolic treatment (i.e. higher dose/particle). The dosimetry selection was based on the mapping study and the patient’s favorable anatomy. We were able to be very selective in the treatment of his oligometastatic disease, with the hypervascularity of the tumors aiding in the protection of his background parenchyma. A single-compartment medical internal radiation dose (MIRD) model was used to calculate radiation dosage due to the minimal amount of involved hepatic parenchyma, with dosing of either 300 Gy or 400 Gy depending on the tumor and size. A partition model with more proximal treatments would have also been possible in this setting, but the elevated lung shunt fraction (12%-16%) would have limited the dose delivered. Particle density ranged from 11,000-52,000 spheres/cm^2^, without any discernible difference in response rates of the lesions based on particle load.

We performed 4 separate highly selective Y90 treatments and thermal ablation of 2 peripheral lesions. The lesions chosen for thermal ablation were easily identified on US and CT, and the mapping showed an unfavorable vascular territory which would dose a significant amount of hepatic parenchyma. Should the patient have recurrent disease in the future, an alternative strategy of embolotherapy will be utilized (e.g., chemoembolization) to avoid any sequelae of cumulative radiation toxicity.

In summary, the super-selective use of Y90 and thermal ablation yielded either complete or partial responses in every lesion treated without significant hepatotoxicity or systemic therapy termination. Currently, the patient is alive without complication from his hepatic metastases treatment, however given his recurrent metastases and residual intracranial disease, he and his family have elected for palliative care. In this select case, meningioma metastases responded well to radiation therapy in the form of Y90. Although it is difficult to draw conclusions based on a single case, the favorable tumor responses and lack of significant adverse events, even in an elderly patient with multiple comorbidities, supports further investigation of an interventional radiology approach to metastatic meningioma treatment.

## Ethics approval and consent to participate

All procedures were performed in accordance with the ethical standards of Weill Cornell’s Medicine’s Instituitional Review Board (IRB) and the 1964 Declaration of Helinski and its later amendments or comparable ethical standards.

## Availability of data and materials

The data used and/or analyzed for this case report are available from the corresponding author on request.

## Author contributions

FP, RM, SP, BL, TS, JK, JI, and BC performed the procedures, image interpretation, pathology interpretation, and pre-/postprocedure follow-up. MF, AH, FP, RM, SP, BL, TS, JK, JI, and BC provided final approval of the submitted manuscript.

## Patient consent

Written informed consent was obtained from the patient for the publication of this case report and any accompanying images.

## References

[1] Tian W., Liu J., Zhao K., Wang J., Jiang W., Shu K. (2020). Analysis of prognostic factors of world health organization grade Ⅲ meningiomas. Front Oncol.

[2] Durand A., Labrousse F., Jouvet A., Bauchet L., Kalamaridès M., Menei P. (2009). WHO grade II and III meningiomas: a study of prognostic factors. J Neurooncol.

[3] Masalha W., Heiland D.H., Delev D., Fennell J.T., Franco P., Scheiwe C. (2019). Survival and prognostic predictors of anaplastic meningiomas. World Neurosurg.

[4] Kunz W.G., Jungblut L.M., Kazmierczak P.M., Vettermann F.J., Bollenbacher A., Tonn J.C. (2017). Improved detection of transosseous meningiomas using 68Ga-DOTATATE PET/CT compared with contrast-enhanced MRI. J Nucl Med.

[5] Ivanidze J., Roytman M., Lin E., Magge R.S., Pisapia D.J., Liechty B. (2019). Gallium-68 DOTATATE PET in the evaluation of intracranial meningiomas. J Neuroimaging.

[6] Perlow H.K., Siedow M., Gokun Y., McElroy J., Matsui J., Zoller W. (2022). 68Ga-DOTATATE PET-based radiation contouring creates more precise radiation volumes for patients with meningioma. Int J Radiat Oncol Biol Phys.

[7] Unterrainer M., Ilhan H., Vettermann F., Cyran C.C., Tonn J.C., Niyazi M. (2019). Whole-body staging of metastatic Atypical meningioma using 68Ga-DOTATATE PET/CT. Clin Nucl Med.

[8] Ivanidze J., Chang S.J., Haghdel A., Kim J.T., Roy Choudhury A., Wu A. (2024). [Ga68] DOTATATE PET/MRI-guided radiosurgical treatment planning and response assessment in meningiomas. Neuro-Oncology.

[9] Kessler R.A., Garzon-Muvdi T., Yang W., Weingart J., Olivi A., Huang J. (2017). Metastatic atypical and anaplastic meningioma: a case series and review of the literature. World Neurosurg.

[10] Chow P.H.W., Gandhi M. (2017). Phase III multi-centre open-label randomized controlled trial of selective internal radiation therapy (SIRT) versus sorafenib in locally advanced hepatocellular carcinoma: the SIRveNIB study. J Clin Oncol.

[11] Salem R., Padia S.A., Lam M., Bell J., Chiesa C., Fowers K. (2019). Clinical and dosimetric considerations for Y90: recommendations from an international multidisciplinary working group. Euro J Nucl Med Mol Imaging.

[12] Kessler R.A., Garzon-Muvdi T., Yang W., Weingart J., Olivi A., Huang J. (2017). Metastatic Atypical and anaplastic Meningioma: A case series and review of the literature. World Neurosurg.

[13] Brastianos P.K., Kim A.E., Giobbie-Hurder A., Lee E.Q., Wang N., Eichler A.F. (2022). Phase 2 study of pembrolizumab in patients with recurrent and residual high-grade meningiomas. Nat Commun.

